# Potassium ditin(IV) tris­[phosphate(V)], KSn_2_(PO_4_)_3_

**DOI:** 10.1107/S1600536811035604

**Published:** 2011-09-14

**Authors:** Hui Ju Zhang

**Affiliations:** a Department of Physics and Chemistry, Henan Polytechnic University, Jiaozuo, Henan 454000, People’s Republic of China

## Abstract

The title compound, KSn_2_(PO_4_)_3_, belongs to the NASICON-type family of phosphates with the space group *R* 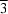. Its structure is constructed by very regular [with P—O distances ranging from 1.513 (6) to 1.522 (6) Å] PO_4_ tetra­hedra and SnO_6_ octa­hedra on the 3. axis, which are linked by O atoms, forming an [Sn_2_(PO_4_)_3_] framework. The K atoms occupy the 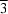. axis sites and are located in the voids of this arrangement. The crystal studied was a merohedral twin with twin law (010 100 00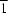) and a component ratio of 0.580 (7):0.420 (7).

## Related literature

For related NASICON-type compounds, see: Alamo & Rodrigo (1992 ▶); Boilot *et al.* (1987 ▶); Boujelben *et al.* (2007 ▶); Zatovskii *et al.* (2006 ▶); Zhao *et al.* (2011 ▶).

## Experimental

### 

#### Crystal data

KSn_2_(PO_4_)_3_
*M* *_r_* = 561.39
Trigonal, 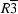

*a* = 8.3381 (1) Å
*c* = 23.5508 (3) Å
*V* = 1417.98 (3) Å^3^
*Z* = 6
Mo *K*α radiation
μ = 6.30 mm^−1^
*T* = 296 K
0.20 × 0.05 × 0.05 mm

#### Data collection

Bruker SMART 1K CCD diffractometer
Absorption correction: multi-scan (*SADABS*; Bruker, 1997 ▶) *T*_min_ = 0.366, *T*_max_ = 0.744
2168 measured reflections
597 independent reflections
591 reflections with *I* > 2σ(*I*)
*R*_int_ = 0.098

#### Refinement

*R*[*F* ^2^ > 2σ(*F* ^2^)] = 0.053
*wR*(*F* ^2^) = 0.137
*S* = 1.24
597 reflections
58 parameters
Δρ_max_ = 2.23 e Å^−3^
Δρ_min_ = −2.99 e Å^−3^

### 

Data collection: *SMART* (Bruker, 1997 ▶); cell refinement: *SMART*; data reduction: *SAINT* (Bruker, 1997 ▶); program(s) used to solve structure: *SHELXS97* (Sheldrick, 2008 ▶); program(s) used to refine structure: *SHELXL97* (Sheldrick, 2008 ▶); molecular graphics: *DIAMOND* (Brandenburg, 2004 ▶); software used to prepare material for publication: *SHELXTL* (Sheldrick, 2008 ▶).

## Supplementary Material

Crystal structure: contains datablock(s) I, global. DOI: 10.1107/S1600536811035604/fi2111sup1.cif

Structure factors: contains datablock(s) I. DOI: 10.1107/S1600536811035604/fi2111Isup2.hkl

Additional supplementary materials:  crystallographic information; 3D view; checkCIF report

## Figures and Tables

**Fig. 1.**
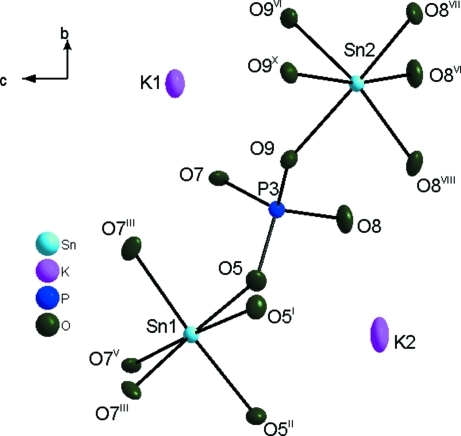
The expanded asymmetric unit of KSn2(PO4)3 showing the coordination environments of the P and Sn atoms. The displacement ellipsoids are drawn at the 50% probability level. [Symmetry codes: (i) -x + y, -x, z; (ii) -y, x-y, z; (iii) y - 1/3, -x + y - 2/3, -z + 1/3; (v) -x - 1/3, -y + 1/3, -z + 1/3; (vi) -x, -y + 1, -z; (vii) x-y + 1, x + 1, -z; (viii) y, -x + y, -z; (x) -y + 1, x-y + 1, z.]

**Fig. 2.**
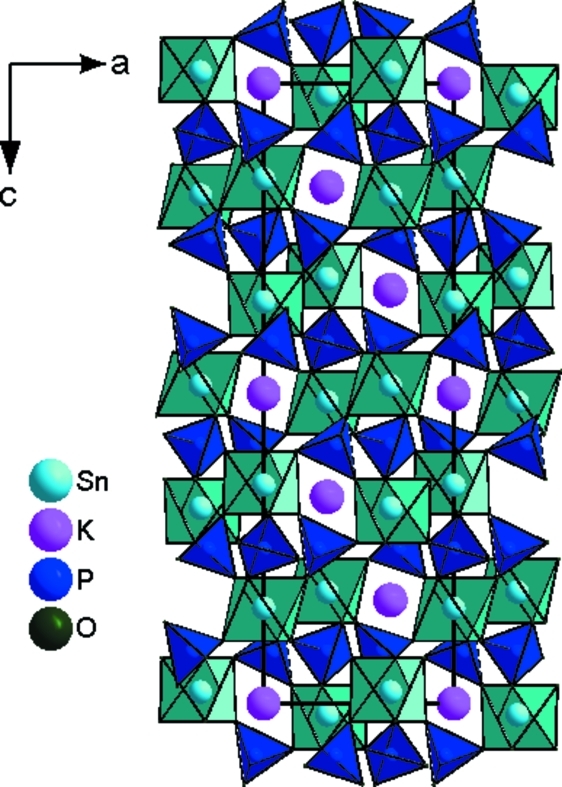
View of the crystal structure of KSn2(PO4)3 along [010]. PO4 and SnO6 units are given in the polyhedral representation.

## supplementary crystallographic information

### Comment

In the past, the family of *AM*_2_(PO_4_)_3_ (*A* = alkali metals; *M* = Ti, Zr, Ge, Sn) compounds with the NASICON-type (Na_3_Zr_2_Si_2_PO_12_: Boilot, *et al.*, 1987) structure have been extensively investigated for their intriguing properities, such as the ionic conductivity properities which may due to the complex and subtle interactions between NASICON framework and mobile ions. The NASICON-type structure with a flexible three-dimensional framework of PO_4_ tetrahedra sharing comers with MO_6_ octahedra, is amenable to a wide variety of chemical substitutions at the various crystallographic positions, thus yielding a large number of closely related compounds, such as NaFeNb(PO_4_)_3_ (Zatovskii, *et al.*, 2006) and K_2_Ca_2_(SO_4_)_3_ (Boujelben, *et al.*, 2007). In order to inrich this family of compounds, we synthesis the compound KSn_2_(PO_4_)_3_ by the high-temperature reaction and determine the crystal structure from single-crystal X-ray diffraction analysis.KSn_2_(PO_4_)_3_ is isostructure with Na (Alamo & Rodrigo, 1992) and Rb (Zhao *et al.*, 2011) analog crystals which crystallizes in the trigonal space group *R*-3.

A projection of the crystal structure of KSn_2_(PO_4_)_3_ is given in Fig. 2. It is characterized by the presence of PO_4_ tetrahedra and SnO_6_ octahedra, linked by sharing corner O atoms, to establish a three-dimentional [Sn_2_(PO_4_)_3_] framework. Furthermore, this framwork delimits two types of channels in which the K atoms are located to compensate the negative charges. The PO_4_ tetrahedra are quite regular, with the P–O distance ranging from 1.513 (6) to 1.522 (6) Å, while the SnO_6_ octahedra is quite regular too, with the Sn–O distance ranging from 2.003 (6) to 2.045 (6) Å.

### Experimental

Compound KSn_2_(PO_4_)_3_ has been prepared by a high-temperature method in air. A powder mixture of K_2_CO_3_, SnO_2_ and NH_4_H_2_PO_4_ in the molar ratio of K: Sn: P = 15: 1: 15 was first ground in an agate mortar and then transferred to a platinum crucible. The sample was gradually heated in air at 1173 K for 24 h. After that, the intermediate product was slowly cooled to 773 K at the rate of 2 K h^-1^, and finally quenched to room temperature. The obtained crystals were colorless with a prismatic shape.

### Refinement

The KSn_2_(PO4)_3_ crystal studies was twinned by merohedry. For refinement the twin law (0 1 0 1 0 0 0 0 1) was used; the twin component ratio refined to 0.580 (7): 0.420 (7). The highest peak in the difference electron density map equals to 2.23 e/Å^3^ at the distance of 0.05 Å from Sn1 site while the deepest hole equals to -2.99 e/Å^3^ at the distance of 0.99 Å from Sn2 site.

### Crystal data

**Table:**

KSn_2_(PO_4_)_3_	*D*_x_ = 3.945 Mg m^−^^3^
*M**_r_* = 561.39	Mo *K*α radiation, λ = 0.71073 Å
Trigonal, *R*3	Cell parameters from 256 reflections
Hall symbol: -R 3	θ = 2.6–23.6°
*a* = 8.3381 (1) Å	µ = 6.30 mm^−^^1^
*c* = 23.5508 (3) Å	*T* = 296 K
*V* = 1417.98 (3) Å^3^	Prism, colourless
*Z* = 6	0.20 × 0.05 × 0.05 mm
*F*(000) = 1560	

### Data collection

**Table:**

Bruker SMART 1K CCD diffractometer	597 independent reflections
Radiation source: fine-focus sealed tube	591 reflections with *I* > 2σ(*I*)
graphite	*R*_int_ = 0.098
\ scans	θ_max_ = 25.7°, θ_min_ = 3.0°
Absorption correction: multi-scan (*SADABS*; Bruker, 1997)	*h* = −10→10
*T*_min_ = 0.366, *T*_max_ = 0.744	*k* = −10→10
2168 measured reflections	*l* = −17→28

### Refinement

**Table:**

Refinement on *F*^2^	Primary atom site location: structure-invariant direct methods
Least-squares matrix: full	Secondary atom site location: difference Fourier map
*R*[*F*^2^ > 2σ(*F*^2^)] = 0.053	*w* = 1/[σ^2^(*F*_o_^2^) + (0.0764*P*)^2^ + 11.5796*P*] where *P* = (*F*_o_^2^ + 2*F*_c_^2^)/3
*wR*(*F*^2^) = 0.137	(Δ/σ)_max_ < 0.001
*S* = 1.24	Δρ_max_ = 2.23 e Å^−^^3^
597 reflections	Δρ_min_ = −2.99 e Å^−^^3^
58 parameters	Extinction correction: *SHELXL97* (Sheldrick, 2008), Fc^*^=kFc[1+0.001xFc^2^λ^3^/sin(2θ)]^-1/4^
0 restraints	Extinction coefficient: 0.0063 (7)

### Special details

**Table:**

Geometry. All e.s.d.'s (except the e.s.d. in the dihedral angle between two l.s. planes) are estimated using the full covariance matrix. The cell e.s.d.'s are taken into account individually in the estimation of e.s.d.'s in distances, angles and torsion angles; correlations between e.s.d.'s in cell parameters are only used when they are defined by crystal symmetry. An approximate (isotropic) treatment of cell e.s.d.'s is used for estimating e.s.d.'s involving l.s. planes.
Refinement. Refinement of *F*^2^ against ALL reflections. The weighted *R*-factor *wR* and goodness of fit *S* are based on *F*^2^, conventional *R*-factors *R* are based on *F*, with *F* set to zero for negative *F*^2^. The threshold expression of *F*^2^ > σ(*F*^2^) is used only for calculating *R*-factors(gt) *etc*. and is not relevant to the choice of reflections for refinement. *R*-factors based on *F*^2^ are statistically about twice as large as those based on *F*, and *R*- factors based on ALL data will be even larger.

### Fractional atomic coordinates and isotropic or equivalent isotropic displacement parameters (Å^2^)

**Table:**

	*x*	*y*	*z*	*U*_iso_*/*U*_eq_	
Sn1	0.0000	0.0000	0.15357 (4)	0.0107 (5)	
Sn2	0.3333	0.6667	0.01858 (4)	0.0103 (5)	
K1	0.3333	0.6667	0.1667	0.0273 (12)	
K2	0.0000	0.0000	0.0000	0.0378 (14)	
P3	−0.0441 (3)	0.3326 (4)	0.08384 (9)	0.0117 (6)	
O5	−0.0732 (11)	0.1435 (9)	0.1006 (2)	0.0174 (15)	
O7	−0.1043 (9)	0.4149 (9)	0.1310 (2)	0.0154 (14)	
O8	−0.1477 (9)	0.3069 (10)	0.0284 (3)	0.0208 (15)	
O9	0.1599 (8)	0.4650 (8)	0.0738 (2)	0.0140 (15)	

### Atomic displacement parameters (Å^2^)

**Table:**

	*U*^11^	*U*^22^	*U*^33^	*U*^12^	*U*^13^	*U*^23^
Sn1	0.0111 (6)	0.0111 (6)	0.0099 (6)	0.0056 (3)	0.000	0.000
Sn2	0.0108 (5)	0.0108 (5)	0.0093 (6)	0.0054 (3)	0.000	0.000
K1	0.0332 (17)	0.0332 (17)	0.015 (2)	0.0166 (9)	0.000	0.000
K2	0.050 (2)	0.050 (2)	0.014 (2)	0.0248 (11)	0.000	0.000
P3	0.0115 (12)	0.0118 (11)	0.0109 (11)	0.0052 (9)	0.0006 (9)	0.0003 (9)
O5	0.024 (4)	0.018 (4)	0.017 (3)	0.015 (3)	0.001 (3)	0.001 (2)
O7	0.023 (3)	0.009 (3)	0.016 (3)	0.009 (3)	0.006 (3)	0.000 (3)
O8	0.020 (4)	0.032 (4)	0.011 (3)	0.013 (3)	−0.003 (3)	0.002 (3)
O9	0.016 (3)	0.014 (3)	0.012 (3)	0.006 (3)	0.000 (2)	−0.003 (2)

### Geometric parameters (Å, °)

**Table:**

Sn1—O5^i^	2.023 (6)	K1—O7^iv^	3.282 (7)
Sn1—O5^ii^	2.023 (6)	K1—O7^xii^	3.282 (7)
Sn1—O5	2.023 (6)	K1—O7^xi^	3.282 (7)
Sn1—O7^iii^	2.032 (6)	K1—O7^x^	3.282 (7)
Sn1—O7^iv^	2.033 (6)	K2—O5	2.853 (6)
Sn1—O7^v^	2.033 (6)	K2—O5^xiii^	2.854 (6)
Sn1—K2	3.6167 (9)	K2—O5^i^	2.854 (6)
Sn2—O8^vi^	2.003 (6)	K2—O5^viii^	2.854 (6)
Sn2—O8^vii^	2.003 (6)	K2—O5^ii^	2.854 (6)
Sn2—O8^viii^	2.003 (6)	K2—O5^xiv^	2.854 (6)
Sn2—O9^ix^	2.045 (6)	K2—O8^xiii^	3.416 (7)
Sn2—O9	2.045 (6)	K2—O8^i^	3.416 (7)
Sn2—O9^x^	2.045 (6)	K2—O8^viii^	3.416 (7)
Sn2—K1	3.4876 (9)	K2—O8^ii^	3.416 (7)
K1—O9^xi^	2.696 (6)	K2—O8^xiv^	3.416 (7)
K1—O9^xii^	2.696 (6)	K2—O8	3.416 (7)
K1—O9^iv^	2.696 (6)	P3—O9	1.513 (6)
K1—O9^ix^	2.696 (6)	P3—O7	1.516 (6)
K1—O9	2.696 (6)	P3—O8	1.520 (6)
K1—O9^x^	2.696 (6)	P3—O5	1.522 (6)
K1—O7^ix^	3.282 (7)	O7—Sn1^v^	2.033 (6)
K1—O7	3.282 (7)	O8—Sn2^vi^	2.003 (6)
			
O5^i^—Sn1—O5^ii^	85.9 (2)	O9—K1—O7^x^	76.59 (17)
O5^i^—Sn1—O5	85.9 (2)	O9^x^—K1—O7^x^	46.76 (15)
O5^ii^—Sn1—O5	85.9 (2)	O7^ix^—K1—O7^x^	113.67 (8)
O5^i^—Sn1—O7^iii^	90.7 (2)	O7—K1—O7^x^	113.67 (8)
O5^ii^—Sn1—O7^iii^	92.1 (2)	O7^iv^—K1—O7^x^	66.33 (8)
O5—Sn1—O7^iii^	176.2 (2)	O7^xii^—K1—O7^x^	66.33 (8)
O5^i^—Sn1—O7^iv^	92.1 (2)	O7^xi^—K1—O7^x^	180.00 (14)
O5^ii^—Sn1—O7^iv^	176.2 (2)	O5—K2—O5^xiii^	122.2 (2)
O5—Sn1—O7^iv^	90.7 (2)	O5—K2—O5^i^	57.8 (2)
O7^iii^—Sn1—O7^iv^	91.2 (2)	O5^xiii^—K2—O5^i^	180.0 (6)
O5^i^—Sn1—O7^v^	176.2 (2)	O5—K2—O5^viii^	122.2 (2)
O5^ii^—Sn1—O7^v^	90.7 (2)	O5^xiii^—K2—O5^viii^	57.8 (2)
O5—Sn1—O7^v^	92.1 (2)	O5^i^—K2—O5^viii^	122.2 (2)
O7^iii^—Sn1—O7^v^	91.2 (2)	O5—K2—O5^ii^	57.8 (2)
O7^iv^—Sn1—O7^v^	91.2 (2)	O5^xiii^—K2—O5^ii^	122.2 (2)
O5^i^—Sn1—K2	51.89 (17)	O5^i^—K2—O5^ii^	57.8 (2)
O5^ii^—Sn1—K2	51.89 (17)	O5^viii^—K2—O5^ii^	180.0 (3)
O5—Sn1—K2	51.89 (17)	O5—K2—O5^xiv^	180.000 (1)
O7^iii^—Sn1—K2	124.44 (17)	O5^xiii^—K2—O5^xiv^	57.8 (2)
O7^iv^—Sn1—K2	124.43 (17)	O5^i^—K2—O5^xiv^	122.2 (2)
O7^v^—Sn1—K2	124.43 (17)	O5^viii^—K2—O5^xiv^	57.8 (2)
O8^vi^—Sn2—O8^vii^	92.4 (2)	O5^ii^—K2—O5^xiv^	122.2 (2)
O8^vi^—Sn2—O8^viii^	92.4 (2)	O5—K2—O8^xiii^	83.25 (18)
O8^vii^—Sn2—O8^viii^	92.4 (2)	O5^xiii^—K2—O8^xiii^	44.79 (16)
O8^vi^—Sn2—O9^ix^	84.6 (3)	O5^i^—K2—O8^xiii^	135.21 (16)
O8^vii^—Sn2—O9^ix^	100.0 (3)	O5^viii^—K2—O8^xiii^	95.98 (17)
O8^viii^—Sn2—O9^ix^	167.3 (3)	O5^ii^—K2—O8^xiii^	84.02 (17)
O8^vi^—Sn2—O9	100.0 (3)	O5^xiv^—K2—O8^xiii^	96.75 (18)
O8^vii^—Sn2—O9	167.3 (3)	O5—K2—O8^i^	96.75 (18)
O8^viii^—Sn2—O9	84.6 (3)	O5^xiii^—K2—O8^i^	135.21 (16)
O9^ix^—Sn2—O9	83.8 (2)	O5^i^—K2—O8^i^	44.79 (16)
O8^vi^—Sn2—O9^x^	167.3 (3)	O5^viii^—K2—O8^i^	84.02 (17)
O8^vii^—Sn2—O9^x^	84.6 (3)	O5^ii^—K2—O8^i^	95.98 (17)
O8^viii^—Sn2—O9^x^	100.0 (3)	O5^xiv^—K2—O8^i^	83.25 (18)
O9^ix^—Sn2—O9^x^	83.8 (2)	O8^xiii^—K2—O8^i^	180.0 (3)
O9—Sn2—O9^x^	83.8 (2)	O5—K2—O8^viii^	84.02 (17)
O8^vi^—Sn2—K1	123.56 (18)	O5^xiii^—K2—O8^viii^	96.75 (18)
O8^vii^—Sn2—K1	123.56 (18)	O5^i^—K2—O8^viii^	83.25 (18)
O8^viii^—Sn2—K1	123.56 (18)	O5^viii^—K2—O8^viii^	44.79 (16)
O9^ix^—Sn2—K1	50.48 (17)	O5^ii^—K2—O8^viii^	135.21 (16)
O9—Sn2—K1	50.48 (17)	O5^xiv^—K2—O8^viii^	95.98 (17)
O9^x^—Sn2—K1	50.48 (17)	O8^xiii^—K2—O8^viii^	116.25 (7)
O9^xi^—K1—O9^xii^	60.9 (2)	O8^i^—K2—O8^viii^	63.75 (7)
O9^xi^—K1—O9^iv^	60.9 (2)	O5—K2—O8^ii^	95.98 (17)
O9^xii^—K1—O9^iv^	60.9 (2)	O5^xiii^—K2—O8^ii^	83.25 (18)
O9^xi^—K1—O9^ix^	119.1 (2)	O5^i^—K2—O8^ii^	96.75 (18)
O9^xii^—K1—O9^ix^	119.1 (2)	O5^viii^—K2—O8^ii^	135.21 (16)
O9^iv^—K1—O9^ix^	179.999 (1)	O5^ii^—K2—O8^ii^	44.79 (16)
O9^xi^—K1—O9	119.1 (2)	O5^xiv^—K2—O8^ii^	84.02 (17)
O9^xii^—K1—O9	179.999 (1)	O8^xiii^—K2—O8^ii^	63.75 (7)
O9^iv^—K1—O9	119.1 (2)	O8^i^—K2—O8^ii^	116.25 (7)
O9^ix^—K1—O9	60.9 (2)	O8^viii^—K2—O8^ii^	180.0 (3)
O9^xi^—K1—O9^x^	179.999 (1)	O5—K2—O8^xiv^	135.21 (16)
O9^xii^—K1—O9^x^	119.1 (2)	O5^xiii^—K2—O8^xiv^	95.98 (17)
O9^iv^—K1—O9^x^	119.1 (2)	O5^i^—K2—O8^xiv^	84.02 (17)
O9^ix^—K1—O9^x^	60.9 (2)	O5^viii^—K2—O8^xiv^	96.75 (18)
O9—K1—O9^x^	60.9 (2)	O5^ii^—K2—O8^xiv^	83.25 (18)
O9^xi^—K1—O7^ix^	103.41 (17)	O5^xiv^—K2—O8^xiv^	44.79 (16)
O9^xii^—K1—O7^ix^	72.93 (16)	O8^xiii^—K2—O8^xiv^	116.25 (7)
O9^iv^—K1—O7^ix^	133.24 (16)	O8^i^—K2—O8^xiv^	63.75 (7)
O9^ix^—K1—O7^ix^	46.76 (16)	O8^viii^—K2—O8^xiv^	116.25 (7)
O9—K1—O7^ix^	107.07 (16)	O8^ii^—K2—O8^xiv^	63.75 (7)
O9^x^—K1—O7^ix^	76.59 (17)	O5—K2—O8	44.79 (16)
O9^xi^—K1—O7	72.93 (16)	O5^xiii^—K2—O8	84.02 (17)
O9^xii^—K1—O7	133.23 (16)	O5^i^—K2—O8	95.98 (17)
O9^iv^—K1—O7	103.41 (17)	O5^viii^—K2—O8	83.25 (18)
O9^ix^—K1—O7	76.59 (17)	O5^ii^—K2—O8	96.75 (18)
O9—K1—O7	46.76 (16)	O5^xiv^—K2—O8	135.21 (16)
O9^x^—K1—O7	107.07 (16)	O8^xiii^—K2—O8	63.75 (7)
O7^ix^—K1—O7	113.68 (8)	O8^i^—K2—O8	116.25 (7)
O9^xi^—K1—O7^iv^	76.59 (17)	O8^viii^—K2—O8	63.75 (7)
O9^xii^—K1—O7^iv^	107.07 (16)	O8^ii^—K2—O8	116.25 (7)
O9^iv^—K1—O7^iv^	46.76 (16)	O8^xiv^—K2—O8	180.0
O9^ix^—K1—O7^iv^	133.24 (16)	O9—P3—O7	106.8 (4)
O9—K1—O7^iv^	72.93 (16)	O9—P3—O8	108.8 (4)
O9^x^—K1—O7^iv^	103.41 (17)	O7—P3—O8	113.5 (4)
O7^ix^—K1—O7^iv^	180.0	O9—P3—O5	109.6 (4)
O7—K1—O7^iv^	66.32 (8)	O7—P3—O5	111.2 (3)
O9^xi^—K1—O7^xii^	107.07 (16)	O8—P3—O5	106.9 (4)
O9^xii^—K1—O7^xii^	46.76 (16)	O9—P3—K1	44.3 (2)
O9^iv^—K1—O7^xii^	76.59 (17)	O7—P3—K1	67.0 (3)
O9^ix^—K1—O7^xii^	103.41 (17)	O8—P3—K1	142.9 (3)
O9—K1—O7^xii^	133.24 (16)	O5—P3—K1	106.8 (3)
O9^x^—K1—O7^xii^	72.93 (16)	O9—P3—K2	88.0 (2)
O7^ix^—K1—O7^xii^	66.32 (8)	O7—P3—K2	160.5 (3)
O7—K1—O7^xii^	180.0	O8—P3—K2	71.8 (3)
O7^iv^—K1—O7^xii^	113.68 (8)	O5—P3—K2	50.5 (2)
O9^xi^—K1—O7^xi^	46.76 (16)	K1—P3—K2	121.08 (7)
O9^xii^—K1—O7^xi^	76.59 (17)	P3—O5—Sn1	146.4 (4)
O9^iv^—K1—O7^xi^	107.07 (16)	P3—O5—K2	105.3 (3)
O9^ix^—K1—O7^xi^	72.93 (16)	Sn1—O5—K2	94.2 (2)
O9—K1—O7^xi^	103.41 (17)	P3—O7—Sn1^v^	136.9 (4)
O9^x^—K1—O7^xi^	133.24 (16)	P3—O7—K1	87.9 (3)
O7^ix^—K1—O7^xi^	66.33 (8)	Sn1^v^—O7—K1	128.9 (2)
O7—K1—O7^xi^	66.33 (8)	P3—O8—Sn2^vi^	151.1 (4)
O7^iv^—K1—O7^xi^	113.67 (8)	P3—O8—K2	83.2 (3)
O7^xii^—K1—O7^xi^	113.67 (8)	Sn2^vi^—O8—K2	124.2 (3)
O9^xi^—K1—O7^x^	133.24 (16)	P3—O9—Sn2	140.7 (4)
O9^xii^—K1—O7^x^	103.41 (17)	P3—O9—K1	112.7 (3)
O9^iv^—K1—O7^x^	72.93 (16)	Sn2—O9—K1	93.7 (2)
O9^ix^—K1—O7^x^	107.07 (16)		

Symmetry codes: (i) −*x*+*y*, −*x*, *z*; (ii) −*y*, *x*−*y*, *z*; (iii) *y*−1/3, −*x*+*y*−2/3, −*z*+1/3; (iv) *x*−*y*+2/3, *x*+1/3, −*z*+1/3; (v) −*x*−1/3, −*y*+1/3, −*z*+1/3; (vi) −*x*, −*y*+1, −*z*; (vii) *x*−*y*+1, *x*+1, −*z*; (viii) *y*, −*x*+*y*, −*z*; (ix) −*x*+*y*, −*x*+1, *z*; (x) −*y*+1, *x*−*y*+1, *z*; (xi) *y*−1/3, −*x*+*y*+1/3, −*z*+1/3; (xii) −*x*+2/3, −*y*+4/3, −*z*+1/3; (xiii) *x*−*y*, *x*, −*z*; (xiv) −*x*, −*y*, −*z*.
